# The Queen's Visit to the Coombe Hospital

**Published:** 1911-07-15

**Authors:** 


### THE KING AND QUEEN IN IRELAND.

### THE QUEEN'S VISIT TO THE COOMBE HOSPITAL.

The Coombe Lying-in Hospital is situated in a very poor part of Dublin, which has come before the public this week as the district in which Lord Iveagh's play-centre, visited by the King, has been established. On Monday her Majesty visited the hospital and had a magnificent reception. The Queen was received by Dr. Gibson, the master, Mr. Wellington Darley, the Chairman of the Committee, Mr. Perry, the Honorary Secretary, Mr. Fred A. Henry, the Registrar, and the following Governors: Rev. J. Dryden Smylie, M.A., Mr. Wm. M. Murphy, J.P., Mr. George Perry, J.P., Very Rev. M. D. Canon Scally, P.P., Mr. W. H. F. Verschoyle, Mr. Michael K. Roche, J.P., Sir Joseph Downes, J.P., Mr. Louis A. Byrne, F.R.C.S.I., Dr. Fred. W. Kidd, Mr. Walter Scott, Mr. John George Fottrell, and the Lord Mayor, who as a director was also present. The medical and surgical staff were represented by the Assistant Masters (Dr. Robert A. MacLaverty and Mr. Louis Cassidy, F.R.C.S.I.), the Supernumerary Assistant Masters (Dr. Thomas Neill and Mr. Victor M'Allister, F.R.C.S.I.), the Consulting Physicians (Sir John William More and Sir Joseph M. Redmond), the Consulting Surgeons (Mr. Francis T. Heuston, F.R.C.S.I., and Dr. Kidd), and the Lady Superintendent, Miss Joy, who carried in her arms a baby born on Coronation Day.

### Her Majesty's Inspection.

The east wing was inspected first, and the Queen not only addressed every patient, but was photographed by the side of one who had recently undergone an operation. The Pembroke Dispensary for infantile diseases was next visited, and from there her Majesty passed to the main building. Here again every patient received a few words, including a lady, Mrs. Owens, of Cardiff, who was occupying one of the private wards. Her Majesty then proceeded to the Board Room, where she signed the visitors' book. Mr. Darley then presented to her Majesty as a souvenir the "Short History of Coombe Hospital, Dublin," which was bound in white morocco, the outside cover being embossed in purple and gold. The end-papers were covered with St. Patrick blue poplin. The text of the history was in wavy black type with red lettering. On leaving her Majesty shook hands with the Master, Mr. Darley, Mr. Henry, the Registrar, and Miss Joy. This visit is comparable only to the inspection which the King made last August of the London Hospital.

